# METABOLIC FLEXIBILITY IN CLASSICAL MONOCYTES IS NOT AFFECTED BY AGE

**DOI:** 10.1093/geroni/igz038.392

**Published:** 2019-11-08

**Authors:** Johnathan Yarbro, Brandt Pence

**Affiliations:** University of Memphis, Memphis, Tennessee, United States

## Abstract

Inflammaging is the chronic low-grade inflammation that occurs with age that contributes to the pathology of age-related diseases. Monocytes are innate immune cells that become dysregulated with age and which can contribute to inflammaging. Metabolism plays a key role in determining immune cell functions, with anti-inflammatory cells primarily relying on fatty acid oxidation and pro-inflammatory cells primarily relying on glycolysis. It was recently shown that lipopolysaccharide (LPS)-stimulated monocytes can compensate for a lack of glucose by utilizing fatty acid oxidation. Given that mitochondrial function decreases with age, we hypothesized that monocytes taken from aged individuals would have an impaired ability to upregulate oxidative metabolism and would have impaired effector functions. Aging did not impair LPS-induced oxygen consumption rate during glucose starvation as measured on a Seahorse XFp system. Additionally, aged monocytes maintained inflammatory gene expression responses and phagocytic capacity during LPS stimulation in the absence of glucose. In conclusion, aged monocytes maintain effector and metabolic functions during glucose starvation, at least in an ex vivo context.

